# Incarceration of a part of the gastric wall into the abdominal cavity in a patient with hiatal hernia and complete dislocation of the stomach (upside‐down stomach)

**DOI:** 10.1002/deo2.377

**Published:** 2024-05-29

**Authors:** Suguru Chiyonaga, Yuki Ohya, Mitsuhiro Inoue, Dan Matsuda, Akira Yoneda, Jun Tomiguchi, Yukari Hinokuma, Shintaro Hayashida, Masayoshi Iizaka, Yukihiro Inomata

**Affiliations:** ^1^ Department of Gastroenterology and Hepatology Kumamoto Rosai Hospital Kumamoto Japan; ^2^ Department of Surgery Kumamoto Rosai Hospital Kumamoto Japan

**Keywords:** Cameron's ulcer, fundoplication, hernia incarceration, hiatal hernia, upside‐down stomach

## Abstract

An upside‐down stomach is a rare type of hiatal hernia. An 83‐year‐old woman presented to the emergency room with abdominal pain and vomiting. Computed tomography revealed an upside‐down stomach and the incarceration of a part of the gastric body into the abdominal cavity. Upper gastrointestinal endoscopy revealed a circular ulcer caused by gastric ischemia. Although she was discharged after 1 week of conservative therapy, she was readmitted to the hospital 1 day after discharge because of a recurrence of hiatal hernia incarceration. She underwent laparoscopic surgery 4 days after readmission and recovered successfully.

## INTRODUCTION

A hiatal hernia is a partial or total dislocation of the stomach through the diaphragmatic esophageal hiatus into the thoracic cavity.^1^, ^2^, ^3^, ^4^ An upside‐down stomach is a rare type of hiatal hernia characterized by herniation of the entire or most of the stomach into the posterior mediastinum.^2^, ^3^, ^4^ In rare cases, patients with an upside‐down stomach have been reported to present with gastric volvulus.^3^, ^4^ However, there are few reports that a part, not a whole, of the stomach incarcerated into the abdominal cavity in patients with upside‐down stomachs.^4^ Herein, we report a case of an upside‐down stomach with the incarceration of a part of the gastric body into the abdominal cavity.

## CASE REPORT

**FIGURE 1:**
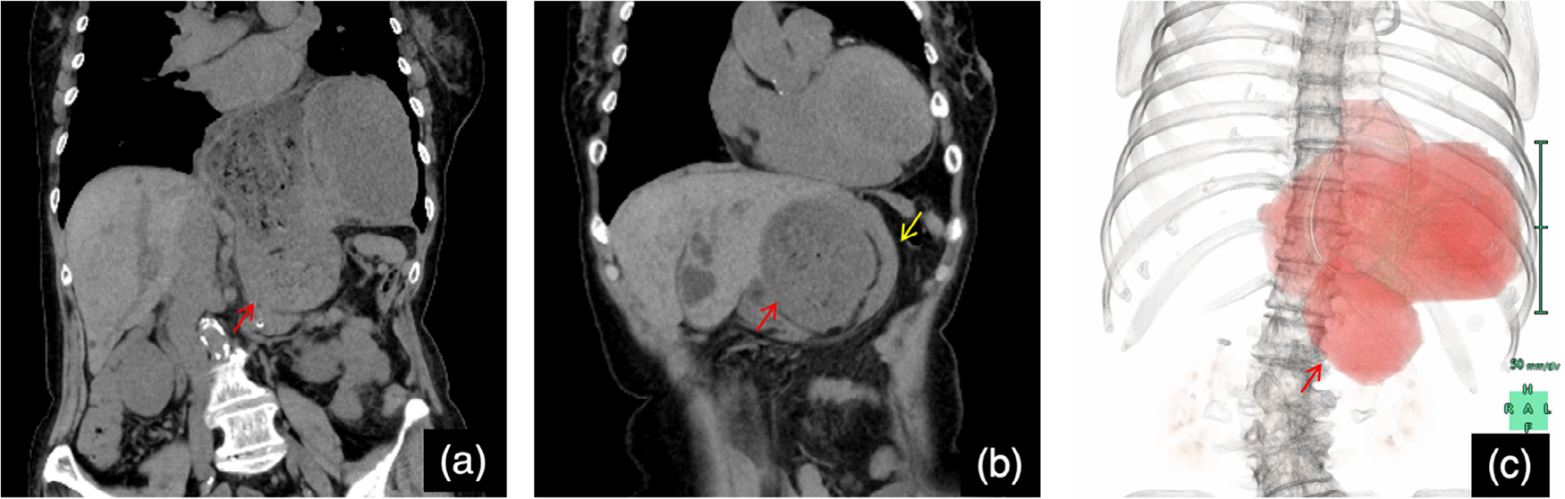
Computed tomography findings on initial admission (a–c) Computed tomography shows hiatal hernia and incarceration of a part of the gastric body into the abdominal cavity. The red arrows show the incarceration of a part of the gastric body into the abdominal cavity, and the yellow arrows show the compressed duodenum.

**FIGURE 2:**
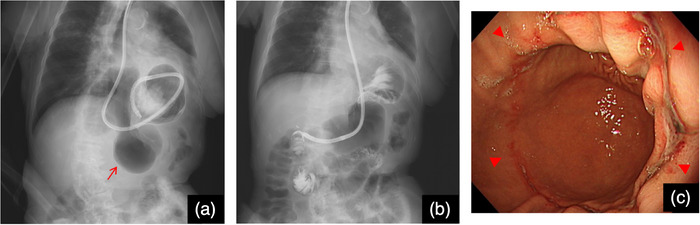
Fluoroscopic and esophagogastroduodenoscopic findings on initial admission (a) This fluoroscopic image shows the incarceration of a part of the gastric body into the abdominal cavity. The red arrow shows the incarceration of a part of the gastric body into the abdominal cavity. (b) This fluoroscopic image shows that the stomach was straightened with esophagogastroduodenoscopy. (c) Esophagogastroduodenoscopy detected Cameron's ulcer. The red arrowheads show the ulcer.

**FIGURE 3:**
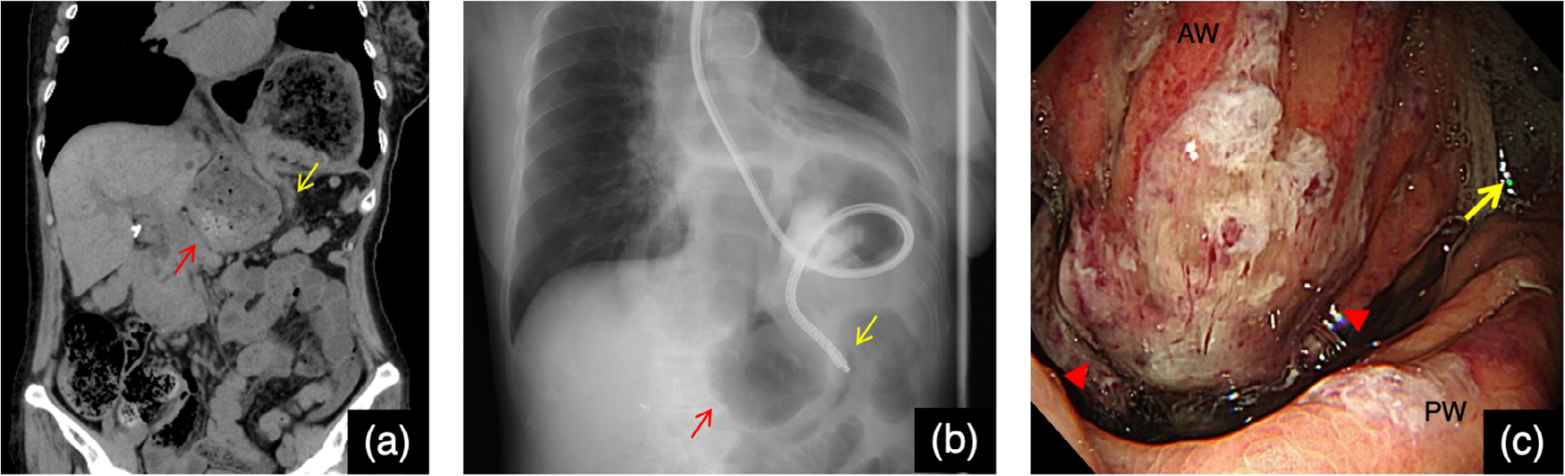
Imaging findings on readmission (a) Computed tomography shows hiatal hernia and incarceration of a part of the gastric body into the abdominal cavity. The red arrow shows the incarceration of a part of the gastric body into the abdominal cavity, and the yellow arrow shows the compressed duodenum. (b) This fluoroscopic image shows the insertion of the esophagogastroduodenoscope into the duodenum to straighten the stomach. The red arrow shows the incarceration of a part of the gastric body into the abdominal cavity, and the yellow arrows show the compressed duodenum. (c) Esophagogastroduodenoscopy detected severe Cameron's ulcer. The red arrowheads show the ulcer. The yellow arrow points in the direction of the gastric antrum. AW: anterior wall, PW: posterior wall.

**FIGURE 4:**
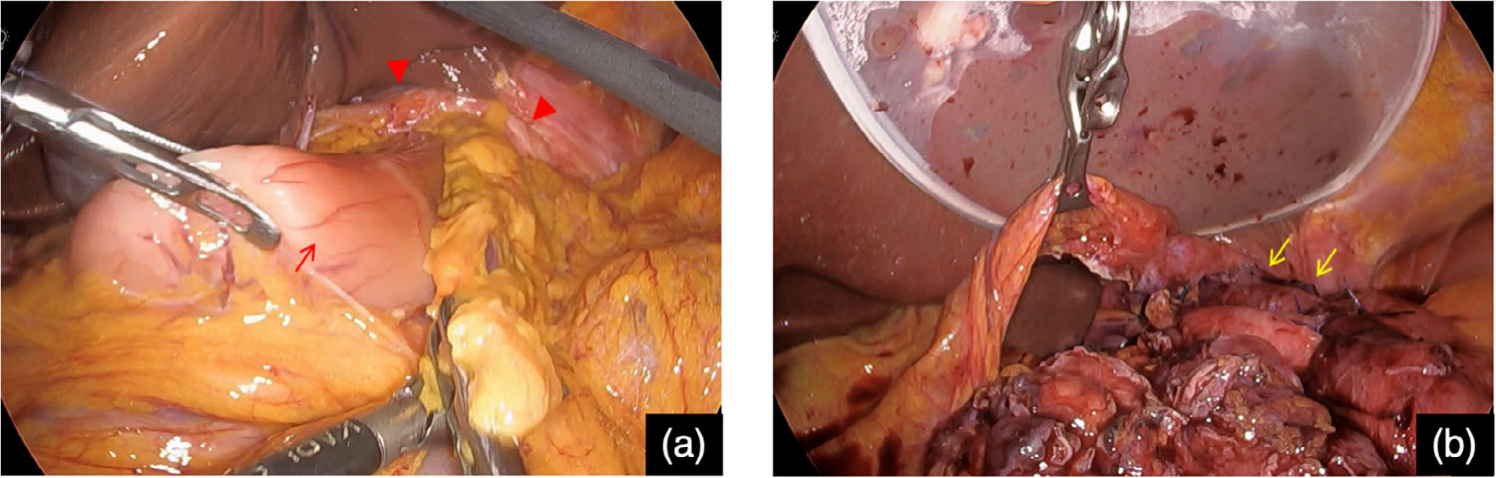
Operative findings (a) The incarceration of a part of the gastric body had been reduced at the time of surgery. The red arrowheads indicate an esophageal hiatus. The red arrow shows the pylorus. (b) The yellow arrows show the fundus cuff created by Toupet fundoplication. The yellow arrow shows Toupet fundoplication.

An 83‐year‐old woman was brought to the emergency room with abdominal pain, nausea, and vomiting. Computed tomography revealed a hiatal hernia and incarceration of a part of the gastric body into the abdominal cavity (Figures 1a–c). She had presented with an asymptomatic upside‐down stomach on computed tomography at least 2 years ago. The patient was admitted to the hospital after the placement of a gastric tube for the drainage of gastric contents. Upper gastrointestinal endoscopy was performed under fluoroscopy the day after admission. The gastric contents were aspirated, and the stomach was straightened (Figures 2a,b). Cameron's ulcer was observed, which presented as a circular and linear ulcer in the gastric body (Figure 2c). After relief from incarceration, the patient's symptoms became milder. She was discharged 7 days later because her symptoms did not deteriorate upon the resumption of eating. However, the day after discharge, abdominal pain and vomiting flared again. She was readmitted after confirmation of the same situation on computed tomography (Figure 3a). After the placement of a gastric tube, another esophagogastroduodenoscopy was conducted under fluoroscopy, and the stomach was straightened (Figure 3b). The second esophagogastroduodenoscopy showed a more severe Cameron's ulcer than the first, with reverse incarceration of a part of the gastric body into the abdominal cavity (Figure 3c). The incarceration was believed to have caused gastric mucosal damage due to gastric ischemia. Due to the recurrence of similar conditions and symptoms despite prior conservative treatment, the patient required surgical treatment. The patient underwent laparoscopic Toupet fundoplication via a transabdominal approach 4 days after readmission (Figures 4a,b). Intraoperative findings revealed an upside‐down stomach. Inverse incarceration of a part of the gastric body was reduced by preoperative conservative treatment (Figure 4a). The crural defect at the esophageal hiatus was closed using interrupted sutures with nonabsorbable braided sutures. The right posterior part of the fundoplication was fixed to the right crus with one nonabsorbable suture. The patient's postoperative period was uneventful, and she was discharged 11 days after surgery. The patient has been doing well without any symptoms 6 months after surgery.

## DISCUSSION

Esophageal hiatal hernias are relatively common, particularly in older people. However, many patients are asymptomatic.^1^ An upside‐down stomach is a rare type of hiatal hernia.^2^, ^3^, ^4^ Gastric volvulus sometimes occurs in patients with an upside‐down stomach.^3^, ^4^ Therefore, Omura et al. reported that surgical treatment should be considered for patients with upside‐down stomachs even if asymptomatic.^4^ They also reported that the outcome of elective repair of paraesophageal hiatus hernia was better than the emergent one.^4^ Since our patient presented with an asymptomatic upside‐down stomach 2 years ago, we should have considered surgical treatment for the patient. However, our conservative treatment with gastric tube insertion and gastroscopy was significant in avoiding emergency surgery.

Although gastric volvulus is reported in patients with an upside‐down stomach, incarceration of a part of the gastric body into the abdominal cavity is even rarer and has not been extensively reported.^5^ In patients with an upside‐down stomach, the entire or most of the stomach is located in the thoracic cavity.^2^, ^3^ The dilatation of the upside‐down stomach caused by food intake causes herniation of part of the gastric body into the abdominal cavity, and this situation can be called “reverse incarceration.”^5^ Although Kuba et al. reported reverse incarceration of a part of the gastric fornix,^5^ our case showed reverse incarceration of a part of the gastric body. The incarcerated stomach in the abdominal cavity compresses the duodenum and makes the stomach more dilated. This vicious cycle worsens the symptoms. As a tentative and conservative treatment, drainage of gastric contents with a gastric tube and straightening of the stomach by esophagogastroduodenoscopy can reduce the incarceration of a part of the gastric body and relieve the outflow blockade. However, surgical intervention is essential to preserve the nutritional status of elderly patients.

In conclusion, patients with an upside‐down stomach may present “reverse incarceration” of the gastric body into the abdominal cavity. Conservative treatment can reduce symptoms, and patients may avoid emergency surgery. However, as in this case, the symptoms may recur after the resumption of eating after conservative treatment. Therefore, patients with an upside‐down stomach and incarceration of a part of the gastric body must undergo surgical treatment after conservative therapy.

## CONFLICT OF INTEREST STATEMENT

None.
